# Fast and Accurate Genome-Wide Association Test of Multiple Quantitative Traits

**DOI:** 10.1155/2018/2564531

**Published:** 2018-03-18

**Authors:** Baolin Wu, James S. Pankow

**Affiliations:** ^1^Division of Biostatistics, School of Public Health, University of Minnesota, Minneapolis, MN, USA; ^2^Division of Epidemiology and Community Health, School of Public Health, University of Minnesota, Minneapolis, MN, USA

## Abstract

Multiple correlated traits are often collected in genetic studies. By jointly analyzing multiple traits, we can increase power by aggregating multiple weak effects and reveal additional insights into the genetic architecture of complex human diseases. In this article, we propose a multivariate linear regression-based method to test the joint association of multiple quantitative traits. It is flexible to accommodate any covariates, has very accurate control of type I errors, and offers very competitive performance. We also discuss fast and accurate significance *p* value computation especially for genome-wide association studies with small-to-medium sample sizes. We demonstrate through extensive numerical studies that the proposed method has competitive performance. Its usefulness is further illustrated with application to genome-wide association analysis of diabetes-related traits in the Atherosclerosis Risk in Communities (ARIC) study. We found some very interesting associations with diabetes traits which have not been reported before. We implemented the proposed methods in a publicly available R package.

## 1. Introduction

Over the past ten years, many epidemiologic studies have used genome-wide association studies (GWAS) to identify genetic components of many complex human diseases. These large cohort studies often collected a broad array of correlated traits that often reflect common physiological processes. By jointly analyzing these correlated traits, we can often gain more power by aggregating multiple weak effects and shed light on the mechanisms underlying complex human diseases [1].

There have been many methods proposed recently to detect SNP association with multiple correlated traits (see, e.g., [2–13]). A direct approach is based on the minimum trait *p* value [6], which typically requires permutations to compute significance *p* value. A related approach is the trait-based association test using an extended Simes procedure (TATES; [10]) that combines the univariate trait *p* values while correcting for the correlations among the multivariate traits. Various dimension reduction methods that summarize the multivariate traits into a univariate outcome are also proposed, which then apply the traditional univariate association test. Examples include the principal component analysis (PCA) [2], principal components of heritability (PCH) [3], and averaging longitudinally observed traits [7, 14]. PCA is an unsupervised dimension reduction and the top PC may not necessarily reflect the association signal. Sample splitting is typically used in PCH for significance calculations and may lead to loss of power.

Multivariate trait testing methods generally perform better than univariate analysis-based approach [15]. Among the multivariate testing methods, a popular approach is the canonical correlation analysis (CCA) [4, 16, 17], which is fast to compute but not flexible and is unable to accommodate covariates. Liu et al. [5] proposed the GEE model [18] to jointly analyze one continuous and one binary trait. In Avery et al. [19] and He et al. [11], GEE-based marginal generalized linear modeling of multivariate traits is adopted for efficient multitrait association testing. Schifano et al. [20] proposed a closely related GEE-based scaled marginal association test of multiple secondary continuous traits. Sitlani et al. [13] explored the GEE modeling of longitudinally measured traits for association test. These GEE-based methods typically explicitly avoided modeling the trait correlations. Another set of multivariate approaches is based on the inverted regression of genotypes to test the overall trait effects. For example, the proportional odds regression modeling of genotypes was proposed as a convenient approach to testing multitrait associations [8, 21, 22]. A related adjacent category logistic regression of genotypes was proposed by Wu and Pankow [12]. Inverted regression approach does not easily accommodate imputed SNPs and has generally used the “best-guess” genotypes, which is known to be leading to a loss of power. In contrast, the multivariate trait regression approach can easily test imputed SNPs by using the imputation dosage as covariate.

In this article, we explore an alternative multivariate regression framework to explicitly model the trait correlation and adjust for covariates to test multitrait associations. We compute the analytical *p* values for the proposed tests based on the *F*-distributions that offer very accurate type I error control with good finite sample performance. We also exploit the parallel nature of genome-wide association test to develop very efficient numerical algorithms that are extremely scalable to genome-wide association tests of millions of SNPs. We demonstrate through extensive numerical studies that the proposed methods have very competitive performance compared to existing methods. We further illustrate the usefulness of the proposed methods through an application to genome-wide association study of multiple diabetes-related glycemic traits.

## 2. Methods

We first discuss a multivariate linear regression-based framework for modeling the multiple quantitative traits and then derive the Wald type statistics for testing multitrait associations.

### 2.1. Multivariate Linear Regression Model

Consider *m* continuous traits *Y* = (*y*_1_,…, *y*_*m*_)^*T*^, a covariate vector *X* = (*x*_1_,…, *x*_*p*_)^*T*^ of length *p* (which could contain an ancestry indicator or principal components), and a genotype score *G* coding the number of minor alleles. Consider the multivariate normal trait model:

(1) $$\begin{array}{c} Y=\beta_{0}+\beta_{X}X+G\beta_{1}+\epsilon , \end{array}$$

where *β*_0_ is a vector of length *m*, *β*_*X*_ is an *m* × *p* matrix, *β*_1_ is a vector of length *m*, and the random error *ϵ* is of length *m* and is assumed to follow a zero mean multivariate normal distribution with covariance Σ, *ϵ* ~ *N*(0, Σ). Multivariate trait association amounts to testing *H*_0_ : *β*_1_ = 0. Here we have assumed the same covariates for all traits, which is the case for our ARIC study GWAS example (see Application to ARIC GWAS of Glycemic Traits) and many typical GWAS. In the supplementary materials (available here), we discuss the possible scenario with different covariates for each trait. The trait model (1) is a multivariate linear model (MLM; see, e.g., [23, chapter 8] and [24, chapter 9]).

Given observations for *n* unrelated individuals, for individual *i*, denote *Y*_*i*_ as the outcome, *X*_*i*_ as the covariate, and *G*_*i*_ as the genotype score. Denote **Y** = (*Y*_1_,…, *Y*_*n*_)^*T*^, **X** = (*X*_1_,…, *X*_*n*_)^*T*^, **G** = (*G*_1_,…, *G*_*n*_)^*T*^, and design matrix **Z** = (1_*n*_, **X**, **G**) of dimension *n* × (*p* + 2), where 1_*n*_ = (1,…, 1)^*T*^ is a column vector of *n* ones.

Denote the *m* × (*p* + 2) parameter matrix **β** = (*β*_0_, *β*_*X*_, *β*_1_). We can check that the maximum likelihood estimators (MLEs) are (see, e.g., [23, p. 294])

(2) β^=YTZZTZ−1,Σ^=1nY−Zβ^TTY−Zβ^T.

### 2.2. Conducting Multivariate Association Tests

Denote the vector operator vec(), which stacks the columns of a matrix into a vector. Denote **A** = **Z**^*T*^**Z**. For the MLEs (2) of the MLM model (1), we can check that (see, e.g., [23, p. 296])

(3) Evecβ^=vecβ,Covvecβ^=A−1⊗Σ,

where ⊗ denotes the Kronecker product and $n\hat{\Sigma }$ independently follows a Wishart distribution, *W*_*m*_(Σ, *n* − *p* − 2), with *n* − *p* − 2 degrees of freedom (DFs) and scale matrix Σ.

Define the *n* × (*p* + 1) design matrix **Z**_0_ = (1_*n*_, **X**) and the corresponding *n* × *n* hat matrix *H* = **Z**_0_(**Z**_0_^*T*^**Z**_0_)^−1^**Z**_0_^*T*^. Let **P** = **I** − *H* and *G*_*e*_ = **P***G*. Here **I** is an *n* × *n* identity matrix. We can check that

(4) β^1=YTGeGeTGe,Covβ^1=GeTGe−1Σ.

We test the multitrait association with the following Wald statistic:

(5) $$\begin{array}{c} Q=\left(\;G_{e}^{T}G_{e}\;\right){\hat{\beta }}_{1}^{T}{\hat{\Sigma }}^{-1}{\hat{\beta }}_{1}. \end{array}$$

Note that ${\hat{\beta }}_{1}$ and $\hat{\Sigma }$ are independent. Under the null hypothesis, ((*n* − *p* − 1 − *m*)/*mn*)*Q* follows the *F*-distribution with (*m*, *n* − *p* − 1 − *m*) DFs (see, e.g., [25, p. 541]).

In the supplementary materials, we analytically show that the CCA test approach [4] is equivalent to a Score test statistic under the MLM model (1) when there are no covariates other than the genotype. Therefore, the proposed MLM-based Wald test can be treated as a natural and flexible generalization of the CCA: (I) it can accommodate any covariates; (II) it is based on the more powerful Wald test instead of the Score test for an association test of quantitative traits; (III) it has an exact *F*-distribution for the multivariate normally distributed traits and hence has very accurate control of type I errors for any sample sizes without the need of asymptotic approximation; and (IV) it is very fast to compute (see next section for details) and extremely scalable to genome-wide association tests of millions of SNPs.

When genetic effects are similar across traits, we can further improve the multivariate association test power using a test statistic with 1-DF following the lines of O'Brien [26], which performed a Wald test of linear combinations of *β*_1_. We can derive similar Wald tests under the MLM (1) (see supplementary materials for technical details). When the genotype effects are the same across different traits, we study the following test statistic:

(6) $$\begin{array}{c} T=\frac{1_{m}^{T}{\hat{\Sigma }}^{-1}{\hat{\beta }}_{1}}{\sqrt{1_{m}^{T}{\hat{\Sigma }}^{-1}1_{m}}}, \end{array}$$

where 1_*m*_ is an *m* × 1 column vector of ones. When the scaled genotype effects are the same across different traits, we study the following test statistic:

(7) $$\begin{array}{c} T^{\prime }=\frac{S^{T}{\hat{\Sigma }}^{-1}{\hat{\beta }}_{1}}{\sqrt{S^{T}{\hat{\Sigma }}^{-1}S}}, \end{array}$$

where *S* is a column vector of estimated standard errors: $S=\sqrt{diag(\hat{\Sigma })}$.

Under the null hypothesis, both *T* and *T*′ follow the asymptotic standard normal distribution. To improve the finite sample performance, we can compare ((*n* − *p* − 1 − *m*)/*n*)*T* and ((*n* − *p* − 1 − *m*)/*n*)*T*′ to a *t*-distribution with (*n* − *p* − 1 − *m*)-DF.

### 2.3. Efficient Computation of GWAS Wald Test Statistics

For a typical GWAS with millions of SNPs, rather than fitting a MLM for each SNP, we developed very efficient algorithm to estimate the MLMs for all SNPs using matrix decomposition tricks following the line of Voorman et al. [27] as follows. For **Z**_0_, denote its singular value decomposition (SVD) as **Z**_0_ = **UDV**^*T*^, where **U** is an *n* × (*p* + 1) matrix with orthogonal columns, **D** is a (*p* + 1)×(*p* + 1) diagonal matrix, and **V** is a (*p* + 1)×(*p* + 1) orthogonal matrix. The null MLM hat matrix can then be computed as *H* = **UU**^*T*^, and *G*_*e*_ = *G* − **U**(**U**^*T*^*G*). Denote the null MLM residual matrix as **E** = **Y** − **U**(**U**^*T*^**Y**), and let **V**_0_ = **E**^*T*^**E**. In (4), we have shown that the genotype effect can be efficiently computed as ${\hat{\beta }}_{1}=Y^{T}G_{e}/(G_{e}^{T}G_{e})$. We can then compute the covariance matrix MLE as $\hat{\Sigma }=V_{0}/n-(G_{e}G_{e}^{T}){\hat{\beta }}_{1}{\hat{\beta }}_{1}^{T}/n$. Here both **V**_0_ and **U** just need to be precomputed once and can be stored for use with all SNPs. Operationally we can also apply the popular PLINK tool [28] to test multitrait association. We first obtain the residuals of multivariate traits and genotypes adjusting for all covariates. We then input the residuals into the PLINK CCA test approach [4]. Technically, we need to adjust the PLINK output *p* value using an *F*-distribution with different DFs (see supplementary materials for technical details).

## 3. Results

### 3.1. Simulation Studies

We consider three forms of Wald statistics: *Q* is the omnibus test, and *T* and *T*′ are the 1-DF test assuming common or common scaled effects. The GEE-based approaches of He et al. [11] are computationally very efficient, have been shown to appropriately control the type I errors, and have the overall best detection power compared to the other methods (e.g., TATES of [10] and other univariate test-based methods) in extensive numerical studies. Here we compared the proposed methods to their GEE score tests, denoted as (*Q*_*s*_, *T*_*s*_, *T*_*s*_′), which are the *m*-DF omnibus test and 1-DF tests assuming a common effect or common scaled effect.

We consider a standard normal covariate *X*_1_ and a Bernoulli covariate *X*_2_ with probability of 0.5. The SNP genotype score *G* is simulated from a Binomial distribution, Binom(2, *f*_0_), where the minor allele frequency (MAF) *f*_0_ = *p*_0_ + *p*_1_*X*_2_. Here *X*_2_ is essentially a population indicator and we have simulated SNPs under population stratification.

We conducted simulations for testing *m* = 2,4, 8 related traits of 1,000 unrelated individuals, respectively. Each time, we simulate the *m* traits from a multivariate normal distribution with a compound symmetry correlation matrix with correlation *ρ*. The first trait has a variance of 2 and all the other traits have unit variance. We set *E*(*Y*_*i*_) = 1 + 0.5*X*_1_ + 0.5*X*_2_ + *γ*_*i*_*G* for *i* = 1,3,…, *m* − 1, and *E*(*Y*_*k*_) = 1 + *X*_1_ + *X*_2_ + *γ*_*k*_*G* for *k* = 2,4,…, *m*.

We used 10 million experiments to evaluate the type I error and 10^5^ experiments to evaluate the power under various combinations of (*γ*_1_,…, *γ*_*m*_). We conducted simulations for *p*_0_ = (0.1,0.3), *p*_1_ = 0.1, and *ρ* = 0,0.2,0.5,0.8. Here we report the results for *m* = 2,8, *ρ* = 0,0.5, and *p*_0_ = 0.1. The conclusions remain the same for other settings (data not shown).

Tables 1 and 2 summarize the estimated type I errors. Overall, the type I errors are well controlled for the proposed methods, while the GEE score tests are conservative, especially for large number of traits (*m* = 8). In general, the proposed Wald tests follow the exact *F*-distribution under the null hypothesis and hence the type I errors are well controlled under all settings. The GEE tests rely on the large-sample asymptotic distribution and therefore generally we need large sample size to have better control of type I errors, especially for a larger number of traits (containing more model parameters).

Tables 3 and 4 summarize the power for *m* = 2 and *m* = 8, respectively. *T* is the most powerful when *γ*_*j*_ are close to each other, and *T*′ is the most powerful when *γ*_*j*_/*σ*_*j*_ are close to each other. In general, the proposed MLM-based Wald tests perform better than the corresponding GEE-based score tests, especially when testing a large number of traits. This agrees with the general principle that the Wald test is typically more powerful than the GEE-based test.

**Figure 1:**
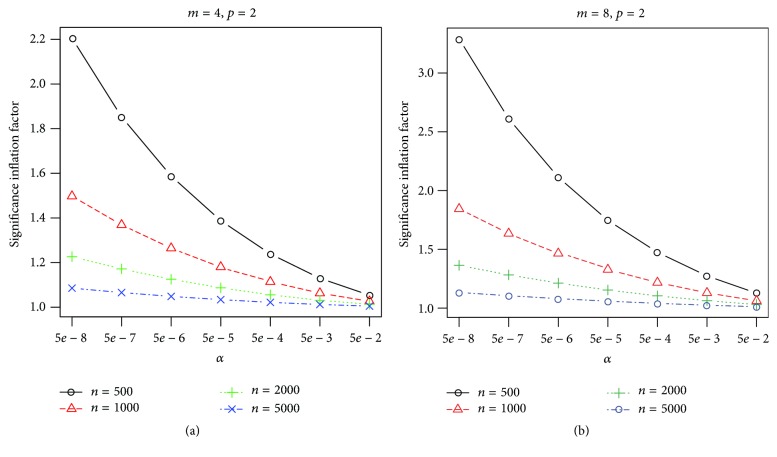
Ratio of the actual significance levels of *m*-DF chi-square test versus the F-test with (*m*, *n* − *p* − 1 − *m*) DFs. The *x*-axis is the type I error rate. (a) Shows the results for testing *m* = 4 traits with *p* = 2 covariates based on *n* individuals. (b) Shows the results for testing *m* = 8 traits with *p* = 2 covariates.

The chi-square statistic ((*n* − *p* − 1)/*n*)*Q* is commonly used in practice and referred to an *m*-DF chi-square distribution to compute multitrait association test's *p* values, which can lead to significantly inflated type I errors at stringent genome-wide significance levels.  Figure 1 shows the ratio of actual significance level of Wald test's *p* values computed using the chi-square distribution and *F*-distribution, respectively. We can see that the type I error based on the chi-square distribution is inflated: more so for larger number of traits, smaller significance level, and smaller sample size. For example, when testing *m* = 8 traits with *p* = 2 covariates and *n* = 500 samples, under genome-wide significance level 5 × 10^−8^, the actual significance level of chi-square distribution *p* value is 3.42 × 5 × 10^−8^ = 1.7 × 10^−7^. Using the chi-square distribution to compute *p* values will lead to very small inflation only when the sample size is large, such as in the meta-analysis of multiple GWAS studies. For typical GWAS with small-to-medium sample sizes, we recommend using the appropriate *F*-distribution to compute significance *p* values to reduce false positive findings.

### 3.2. Application to ARIC GWAS of Glycemic Traits

The Atherosclerosis Risk in Communities (ARIC) study [29] is a population-based, multicenter prospective investigation of cardiovascular disease. Men and women aged 45–64 years at baseline were recruited from four US communities: Forsyth County, North Carolina; Jackson, Mississippi; suburban areas of Minneapolis, Minnesota; and Washington County, Maryland. A total of 15,792 individuals participated in the baseline examination during the period of 1987–1989. The vast majority of ARIC participants are of European (73%) or African (26%) ancestry. We conducted two analyses of diabetes-related glycemic traits in ARIC GWAS data, which has been imputed to around 2.5 million HapMap SNPs using MaCH [30]. We included in the analysis those common SNPs with MAF ≥0.05 and imputation score *R*^2^ ≥ 0.3.

As a proof of concept, we first analyzed four fasting glucose levels in 5947 nondiabetic ARIC white participants measured at four visits (visits 1–4) conducted approximately three years apart. The average correlation of glucose levels is 0.55. We applied an additive genetic model with imputed dosage as a covariate and adjusted for age, gender, and study center in all tests. By analyzing four fasting glucose measures jointly, *T*′ identified 104 significant SNPs, *T* identified 103, *T*_*s*_′ identified 102, *T*_*s*_ identified 101, and *Q* and *Q*_*s*_ identified the same set of 95 SNPs at the genome-wide significance level 5 × 10^−8^. Analyzing each glucose measure separately identified 34, 84, 37, and 64 genome-wide significant SNPs at visits 1, 2, 3, and 4, respectively. All the identified SNPs by different methods are genome-wide significant in the MAGIC Consortium, a meta-analysis of 21 fasting glucose GWAS which together included 46,186 nondiabetic participants [31].

Compared to *T*_*s*_′, the two additional SNPs identified by *T*′, rs780093 and rs780094, had *p* values of 4.8 × 10^−8^ and 4.8 × 10^−8^ using *T*′. Their respective MAGIC meta-analysis' *p* values were 2.9 × 10^−13^ and 2.5 × 10^−12^. Compared to *T*_*s*_, the two additional SNPs identified by *T*, rs1260326 and rs11688384, had *p* values of 4.7 × 10^−8^ and 4.0 × 10^−8^ using *T*. Their respective MAGIC meta-analysis' *p* values were 4.3 × 10^−13^ and 4.1 × 10^−10^.

Second, we jointly analyzed three distinct diabetes-related glycemic traits measured at visit 4 in 5068 nondiabetic white participants measured at visit 4 in ARIC: fasting glucose, fasting insulin, and glucose level 2 hours after an oral glucose challenge. We applied an additive genetic model with imputed dosage as a covariate and adjusted for age, gender, and study center. To account for the skewed distribution of fasting insulin, we adopted the Box-Cox transformation with an estimated power of 0.35 [32]. The three traits had an average pairwise correlation of 0.31. When analyzing fasting insulin or 2-hour glucose levels individually, we did not identify any significant SNPs at the genome-wide significance level (5 × 10^−8^). For joint testing of all three traits, *T*_*s*_, *T*_*s*_′, *T*, *T*′ identified none, *Q*_*s*_ identified 139, and *Q* identified 140 genome-wide significant SNPs, among which 61 and 61 SNPs were reported as genome-wide significant in the MAGIC meta-analyses of fasting glucose, fasting insulin, or 2-hour glucose levels [31, 33].

Compared to *Q*_*s*_, *Q* identified two additional genome-wide significant SNPs, rs4665987 and rs853780, with *p* values of 4.9 × 10^−8^ and 4.9 × 10^−8^, respectively. MAGIC meta-analysis of fasting glucose reported a *p* value of 2.1 × 10^−38^ for rs853780. Its MAGIC meta-analyses of fasting insulin and 2-hour glucose *p* values are 0.054 and 0.477, respectively. For rs4665987 (near GCKR on chromosome 2:27755825), MAGIC meta-analysis' *p* values for the fasting glucose, fasting insulin, and 2-hour glucose levels are 4.6 × 10^−6^, 0.04, and 9.3 × 10^−5^, respectively. This SNP was genome-wide significantly associated with human serum metabolite levels in a GWAS of 8330 Finnish individuals [34] and several other GWAS [35–38]. Compared to *Q*, *Q*_*s*_ reported one additional genome-wide significant SNP, rs17540154, with *p* value of 4.3 × 10^−8^. The MAGIC meta-analysis of fasting glucose reported a *p* value of 8.7 × 10^−38^ for rs17540154. Its MAGIC meta-analyses of fasting insulin and 2-hour glucose *p* values are 0.101 and 0.720, respectively.

Among the identified significant SNPs by joint testing, there were 79 novel genome-wide significant SNPs that have not been reported as significantly associated with diabetes-related fasting glucose and insulin levels before. Among them, one SNP, rs4665987, is located on chromosome 2:27755825 and 78 other SNPs are clustered on chromosomes 15:62132921 to 15:62396389. Interestingly, six of them (listed in Table 5) were genome-wide significant in the MAGIC meta-analysis of proinsulin level [39]. The list of all identified SNPs with detailed analysis' results is available in the supplementary materials.

## 4. Discussion

So far typical effect sizes of most identified genetic variants for many diseases or traits are very small and they have only explained a very small proportion of the overall disease heritability or trait variation. It is commonly accepted that there are many more common variants with relatively small-to-medium effect sizes or rare variants with larger effect sizes yet to be discovered. To identify these additional variants, very large sample sizes will be needed. One approach is to form a consortium to facilitate meta-analysis of many studies, but development of these genetics consortia is generally time-consuming and logistically challenging. Meanwhile the recently studied joint association test of multiple correlated traits offers an alternative approach to boost power in that it can often dramatically improve the association test power by “enlarging the sample size” through the incorporation of many correlated traits that are typically collected in most large genetic studies and may share genetic determinants. Another strategy to further improve the detection power is to use a variant-set association test, which has been proven to be very useful (see, e.g., [16, 17, 40–42]). It is worthwhile to generalize the proposed Wald tests to develop more accurate and powerful association tests of variant sets across multiple traits.

Here we have focused on testing a relatively small number of correlated quantitative traits, which have enabled us to develop accurate and powerful association tests without any asymptotic approximations as adopted in the more general though conservative GEE approach, which can be applied to any mix of quantitative and discrete traits. It will be interesting to extend the proposed methods to the phenome-wide association studies (PheWAS) with a large collection of phenotypes [43–45] and develop more powerful joint association test of quantitative and discrete traits.

In the previous discussions, we have assumed the same set of covariates across all traits. With differing covariates, we provide technical details regarding model estimation and extensive simulation studies to confirm that the proposed methods accurately control type I errors and perform favorably compared to existing methods (see the supplementary materials for complete results). In summary, we recommend the proposed multivariate linear regression-based test as a complementary approach to enhancing the power of analyzing multiple quantitative traits in unrelated individuals. Our numerical studies have suggested that the omnibus Wald test generally has robust and good performance. The 1-DF Wald tests can perform well due to reduced DFs, but they could be sensitive to the underlying assumptions. It will be worthwhile to develop adaptive and powerful tests. We have implemented the proposed methods in an R package available at http://www.github.com/baolinwu/MTAR. We provide some sample R codes to install and use the package in the supplementary materials. The developed algorithms are very efficient and extremely scalable to genome-wide association test.

## Figures and Tables

**Table 1:** Type I error of multitrait tests (*m* = 2, *p*_0_ = 0.1) divided by the nominal significance level *α*. The MAFs of SNP are 0.1 and 0.2 in the two populations, respectively. *Q* is the *m*-DF omnibus Wald test; *T* and *T*′ are the 1-DF Wald tests assuming a common or common scaled effect. (*Q*_*s*_, *T*_*s*_, *T*_*s*_′) are the corresponding GEE-based *m*-DF omnibus test and 1-DF tests assuming a common effect or common scaled effect.

*α*	*ρ* = 0			*ρ* = 0.5		
	10^−5^	10^−4^	10^−3^	10^−5^	10^−4^	10^−3^
*Q*_*s*_	0.69	0.79	0.89	0.67	0.79	0.89
*T*_*s*_	0.74	0.85	0.93	0.71	0.83	0.92
*T*_*s*_′	0.74	0.85	0.89	0.71	0.83	0.92
*Q*	1.04	1.00	1.00	1.03	1.01	1.00
*T*	0.98	0.99	1.01	0.97	0.99	1.00
*T*′	0.96	0.98	1.00	0.96	0.98	0.99

**Table 2:** Type I error divided by the nominal significance level *α* for multitrait tests (*m* = 8, *p*_0_ = 0.1).

*α*	*ρ* = 0			*ρ* = 0.5		
	10^−5^	10^−4^	10^−3^	10^−5^	10^−4^	10^−3^
*Q*_*s*_	0.43	0.62	0.75	0.44	0.60	0.75
*T*_*s*_	0.74	0.84	0.93	0.77	0.85	0.93
*T*_*s*_′	0.74	0.84	0.93	0.78	0.85	0.93
*Q*	0.94	0.99	1.00	0.94	1.00	1.00
*T*	1.03	1.03	1.02	1.05	1.04	1.03
*T*′	1.03	1.03	1.03	1.03	0.99	0.99

**Table 3:** Power of multitrait tests for *m* = 2 continuous traits (*Y*_1_, *Y*_2_) under significance level *α* = 10^−4^. The MAFs of SNP are 0.1 and 0.2 in the two populations, respectively. *Q* is the *m*-DF omnibus Wald test; *T* and *T*′ are the 1-DF Wald tests assuming common or common scaled effect. (*Q*_*s*_, *T*_*s*_, *T*_*s*_′) are the corresponding GEE-based *m*-DF omnibus test and 1-DF tests assuming a common effect or common scaled effect. *σ*_*i*_ is the standard error of *Y*_*i*_ and *γ*_*i*_ is the SNP coefficient, *i* = 1,2. The highest powered tests are bold-faced.

(*γ*_1_, *γ*_2_)	${\left(\frac{\gamma_{1}}{\sigma_{1}},\frac{\gamma_{2}}{\sigma_{2}}\right)}^{}$	*Q*	*T*	*T*′	*Q*_*s*_	*T*_*s*_	*T*_*s*_′
*ρ* = 0.5							

(0.3,0)	(0.21,0)	**0.375**	0.001	0.024	0.334	0.001	0.019
(0.3,0.1)	(0.21,0.1)	**0.206**	0.047	0.146	0.177	0.039	0.126
(0.25,0.18)	(0.18,0.18)	0.180	0.221	**0.258**	0.154	0.194	0.233
(0.3,0.25)	(0.21,0.25)	0.523	0.617	**0.619**	0.476	0.573	0.582
(0.2,0.2)	(0.14,0.2)	0.179	**0.257**	0.215	0.154	0.23	0.193
(0.2,0.25)	(0.14,0.25)	0.410	**0.501**	0.369	0.367	0.466	0.333
(0.25,0.25)	(0.18,0.25)	0.449	**0.560**	0.492	0.403	0.521	0.455
(0,0.25)	(0,0.25)	**0.638**	0.278	0.052	0.59	0.247	0.040
(0,0.3)	(0,0.3)	**0.893**	0.525	0.121	0.865	0.477	0.093
(0.1,0.25)	(0.07,0.25)	0.465	**0.485**	0.372	0.418	0.448	0.330
(0.1,0.3)	(0.07,0.3)	**0.744**	0.726	0.590	0.700	0.688	0.534
(0.2,0.3)	(0.14,0.3)	0.845	**0.891**	0.842	0.810	0.870	0.810

*ρ* = 0							

(0.3,0)	(0.21,0)	**0.206**	0.026	0.063	0.178	0.020	0.051
(0.3,0.1)	(0.21,0.1)	0.316	0.249	**0.337**	0.278	0.215	0.304
(0.25,0.18)	(0.18,0.18)	0.419	0.510	**0.530**	0.376	0.471	0.494
(0.3,0.25)	(0.21,0.25)	0.830	0.891	**0.892**	0.796	0.868	0.870
(0.2,0.2)	(0.14,0.2)	0.375	**0.486**	0.462	0.333	0.449	0.427
(0.2,0.25)	(0.14,0.25)	0.631	**0.727**	0.677	0.584	0.692	0.636
(0.25,0.25)	(0.18,0.25)	0.734	**0.820**	0.801	0.690	0.792	0.771
(0,0.25)	(0,0.25)	**0.405**	0.249	0.134	0.36	0.217	0.107
(0,0.3)	(0,0.3)	**0.701**	0.485	0.29	0.657	0.437	0.235
(0.1,0.25)	(0.07,0.25)	**0.451**	0.385	0.165	0.406	0.356	0.140
(0.1,0.3)	(0.07,0.3)	**0.769**	0.639	0.301	0.728	0.605	0.257
(0.2,0.3)	(0.14,0.3)	0.700	**0.743**	0.545	0.655	0.713	0.500

**Table 4:** Power of multitrait tests for *m* = 8 continuous traits under significance level *α* = 10^−4^. The MAFs of SNP are 0.1 and 0.2 in the two populations, respectively. *Q* is the *m*-DF omnibus Wald test; *T* and *T*′ are the 1-DF Wald tests assuming common or common scaled effect. (*Q*_*s*_, *T*_*s*_, *T*_*s*_′) are the corresponding GEE-based *m*-DF omnibus test and 1-DF tests assuming a common effect or common scaled effect. The highest powered tests are bold-faced.

(*γ*_1_,…, *γ*_8_)	*Q*	*T*	*T*′	*Q*_*s*_	*T*_*s*_	*T*_*s*_′
*ρ* = 0.5						

*γ*_1_ = 0.3, *γ*_*i*>1_ = 0	**0.303**	0.001	0	0.229	0	0
(.3, .2, .1, .05,0,…, 0)	**0.696**	0	0.008	0.599	0	0.005
*γ*_1_ = 0.2, *γ*_*i*>1_ = 0.15	0.045	0.201	**0.220**	0.030	0.169	0.195
*γ*_*i*_ = 0.15	0.048	**0.237**	0.193	0.032	0.204	0.170

*ρ* = 0						

*γ*_1_ = 0.3, *γ*_*i*>1_ = 0	**0.063**	0.001	0.004	0.043	0.001	0.002
(.3, .2, .1, .05,0,…, 0)	**0.467**	0.156	0.224	0.372	0.102	0.152
*γ*_1_ = 0.2, *γ*_*i*>1_ = 0.15	0.934	0.996	**0.997**	0.887	0.992	0.993
*γ*_*i*_ = 0.15	0.912	**0.995**	0.994	0.855	0.989	0.988

**Table 5:** Six novel SNPs identified in the ARIC joint association test, which were not significant in the corresponding MAGIC consortium meta-analyses of fasting glucose (FG), fasting insulin (FI), and 2-hour fasting glucose (2hFG) but were significant in the MAGIC meta-analysis of fasting proinsulin (FP). We listed the ARIC joint test's *p* values (the proposed MLM Wald test and the GEE chi-square test) and the corresponding MAGIC consortium meta-analyses' *p* values for FG, FI, 2hFG, and FP.

SNP	Chr	bp	ARIC joint test's *p* value		MAGIC meta-analysis' *p* value			
			Wald	GEE	FG	FI	2hFG	FP
rs4502156	15	62383155	5.4*E* − 09	7.9*E* − 09	8.4*E* − 08	6.7*E* − 01	8.2*E* − 05	3.8*E* − 11
rs7163757	15	62391608	1.4*E* − 08	1.8*E* − 08	4.2*E* − 07	5.7*E* − 01	1.9*E* − 05	3.9*E* − 11
rs8037894	15	62394264	1.2*E* − 08	1.6*E* − 08	4.1*E* − 07	4.8*E* − 01	3.5*E* − 05	8.7*E* − 11
rs6494307	15	62394690	1.7*E* − 08	2.1*E* − 08	3.3*E* − 07	4.9*E* − 01	2.7*E* − 05	4.1*E* − 11
rs7167878	15	62396189	1.7*E* − 08	2.1*E* − 08	4.6*E* − 07	4.5*E* − 01	2.4*E* − 05	4.1*E* − 11
rs7172432	15	62396389	1.7*E* − 08	2.2*E* − 08	6.5*E* − 07	3.3*E* − 01	1.9*E* − 05	4.3*E* − 11

## Supplementary Materials

Supplementary Materials In the supplementary materials, we provide more details for (1) model estimation and inference with numerical results when we have different covariates for each trait, (2) 1-DF Wald test assuming similar effects across all traits, (3) relation of proposed methods to CCA (e.g., as implemented in the PLINK), (4) the list of all identified SNPs with detailed analysis results for the ARIC GWAS of glycemic traits, (5) sample codes to install and use the developed R package, and (6) simulation results illustrating the robustness of proposed tests to deviation from normality and impact of directions of marginal effects and trait correlations on the multitrait association test power.
