# Supplementary material for: Fast and Accurate Genome-Wide Association Test of Multiple Quantitative Traits
Source: Comput Math Methods Med. 2018 Mar 18;2018:2564531. doi: 10.1155/2018/2564531 (PMC5878919; doi:10.1155/2018/2564531)
Supplement: Supplementary Materials — In the supplementary materials, we provide more details for (1) model estimation and inference with numerical results when we have different covariates for each trait, (2) 1-DF Wald test assuming similar effects across all traits, (3) relation of proposed methods to CCA (e.g., as implemented in the PLINK), (4) the list of all identified SNPs with detailed analysis results for the ARIC GWAS of glycemic traits, (5) sample codes to install and use the developed R package, and (6) simulation results illustrating the robustness of proposed tests to deviation from normality and impact of directions of marginal effects and trait correlations on the multitrait association test power. [file 2564531.f1.pdf]

Supplementary material for "Fast and accurate genome-wide association test of multiple continuous traits"

Baolin Wu[1], James S. Pankow[2]

[1]Division of Biostatistics, [2]Division of Epidemiology and Community Health

School of Public Health, University of Minnesota

1 CCA as a score test under MLM

When there are no covariates, CCA is typically applied to test the multi-trait association. Here we show that CCA is equivalent to a score statistic under the MLM. Without loss of generality, assume the genotype and individual trait have been centered. For sample $i = 1, \ldots, n$, consider

$$y_{ki} = g_i \beta_k + \epsilon_{ki}, \quad k = 1, \ldots, m,$$

where the error vector $(\epsilon_{1i}, \ldots, \epsilon_{mi})^T$ follows a zero-mean multivariate normal distribution with covariance matrix Σ, where variance $Var(\epsilon_{ki}) = \sigma_k^2$ and covariance $Cov(\epsilon_{ki}, \epsilon_{ji}) = \sigma_{kj}$. Denote $Y_i = (y_{1i}, \ldots, y_{mi})^T$, $\boldsymbol{Y} = (Y_1, \ldots, Y_n)$, $G = (g_1, \ldots, g_n)^T$, and $\boldsymbol{\beta} = (\beta_1, \ldots, \beta_m)^T$.

The log likelihood is proportional to

$$\ell = -n \log |\Sigma| - \sum_{i=1}^{n} (Y_i - g_i \boldsymbol{\beta})^T \Sigma^{-1} (Y_i - g_i \boldsymbol{\beta}).$$

Hence we can easily show that the score vector for testing $H_0 : \boldsymbol{\beta} = 0$ is

$$U = \sum_{i=1}^{n} g_i \Sigma^{-1} Y_i = \Sigma^{-1}(\boldsymbol{Y} G) = (G^T \otimes \Sigma^{-1}) vec(\boldsymbol{Y}),$$

where $\otimes$ means the matrix Kronecker product, and $vec()$ is the vector operator which stacks the columns of a matrix into a column vector. Note $Cov[vec(\boldsymbol{Y})] = \boldsymbol{I} \otimes \Sigma$. Hence

$$Cov(U) = (G^T G) \otimes \Sigma^{-1} = n S_{11} \Sigma^{-1},$$

where $S_{11} = G^T G/n$ is the sample variance of genotype. Denote $S_{21} = \boldsymbol{Y} G/n$, which is the sample covariance vector of multi-traits and genotype. The multi-trait association can be based on the following chi-square statistic, $U^T \widehat{Cov}(U)^{-1} U$, which is

$$Q = n \frac{S_{21}^T S_{22}^{-1} S_{21}}{S_{11}},$$

where we have plugin the estimated null covariance matrix $\hat{\Sigma}$ using the sample covariance matrix S_{22} of multi-traits. The CCA test statistic used in Ferreira and Purcell (2009) is then

$$\frac{Q/n}{1 - Q/n} \frac{n - m - 1}{m}.$$

Therefore the proposed MLM based Wald test can be treated as a natural and flexible generalization of the CCA: (1) it can accommodate any covariates; (2) it is based on the more powerful Wald test instead of the Score test for association tests of quantitative

traits; and (3) it has an exact F-distribution for the multivariate normally distributed multiple continuous traits, and hence has very accurate control of type I errors.

It is not hard to verify that previous derivations still hold when we replace $\boldsymbol{Y}$ and G by their residuals regressing on a common set of p covariates. Therefore operationally we can apply the popular PLINK tool (Purcell *et al.*, 2007) to test multi-trait association as follows. We first obtain the residuals of multivariate traits and genotypes adjusting for all covariates. We then input the residuals into the CCA test approach (Ferreira and Purcell, 2009) implemented in PLINK. Technically we need to adjust the PLINK output p-value T using a F-distribution with different DFs as $1-F_{m,n-m-1-p}(F^{-1}_{m,n-m-1}(1-T)\frac{n-m-1-p}{n-m-1})$, where $F_{d_1,d_2}(\cdot)$ is the distribution function of F-distribution with (d_1,d_2) DFs. Note that when the set of covariates are independent of the genotypes (e.g., age and gender), we can directly use the genotypes instead of the genotype residuals, which can further save some computation time.

2 Multivariate trait association detection using the 1-DF Wald test

Consider the linear combination $U = a^T\hat{\beta}_1$, which follows a normal distribution, $U \sim N(a^T\gamma, (G_e^TG_e)^{-1}a^T\Sigma a)$, where γ is the true value of β_1. Assuming a common genotype effect across the multivariate traits, we have $\gamma = \eta\mathbf{1}_m$. The effect size of U is then proportional to

$$\frac{\eta(a^T\mathbf{1}_m)}{\sqrt{(G_e^TG_e)^{-1}a^T\Sigma a}} = \eta\sqrt{G_e^TG_e}b^T\Sigma^{-1/2}\mathbf{1}_m, \quad b = \frac{\Sigma^{1/2}a}{\sqrt{a^T\Sigma a}}.$$

Taking $b \propto \Sigma^{-1/2}\mathbf{1}_m$ will maximize the effect size (note $b^T b = 1$). Therefore we use the following statistic

$$T = \frac{\mathbf{1}_m^T \hat{\Sigma}^{-1}\hat{\beta}_1}{(\mathbf{1}_m^T \hat{\Sigma}^{-1}\mathbf{1}_m)^{1/2}}.$$

With a common scaled genotype effect across the multivariate traits, we have $\gamma = \eta S$, where $S = (s_1, \cdots, s_m)^T$ with $s_k = \sqrt{\Sigma_{kk}}$, $k = 1, \cdots, m$. Similarly we can derive the following test statistic

$$T' = \frac{S^T \hat{\Sigma}^{-1}\hat{\beta}_1}{(S^T \hat{\Sigma}^{-1} S)^{1/2}}.$$

3 Chi-square and F-distribution based p-value calculation

The chi-square statistic $\frac{n-p-1}{n}Q$ is commonly used in practice and referred to a m-DF chi-square distribution to compute multi-trait association test p-values, which can lead to significantly inflated type I errors at stringent genome-wide significance levels. Figure 1 shows the ratio of actual significance level of Wald test p-values computed using the chi-square distribution and F-distribution respectively. We can see that the type I error based on the chi-square distribution is inflated: more so for larger number of traits, smaller significance level, and smaller sample size. For example, when testing $m = 8$ traits with $p = 2$ covariates and $n = 500$ samples, under genome-wide significance level 5×10^{-8}, the actual significance level of chi-square distribution p-value is $3.42 \times 5 \times 10^{-8} = 1.7 \times 10^{-7}$. Using the chi-square distribution to compute p-values will lead to very small inflation only when the sample size is large, such as in the meta-analysis of multiple GWAS studies. Figure 2 shows the minimum sample size required to have the type I error inflation ≤ 1.1 at significance level $\alpha = 5 \times 10^{-8}$ as a function of number of traits m, when using the chi-square distribution instead of F-distribution to compute p-values. The minimum sample

Figure 1: Nominal significance level inflation factor (IF): plotted are the actual significance level ratios of m-DF chi-square test versus the F-test with $(m, n-p-1-m)$ DFs. The x-axis is the Type I error rate. The left panel shows the results for testing $m = 4$ traits with $p = 2$ covariates based on n individuals. The right panel shows the results for testing $m = 8$ traits with $p = 2$ covariates.

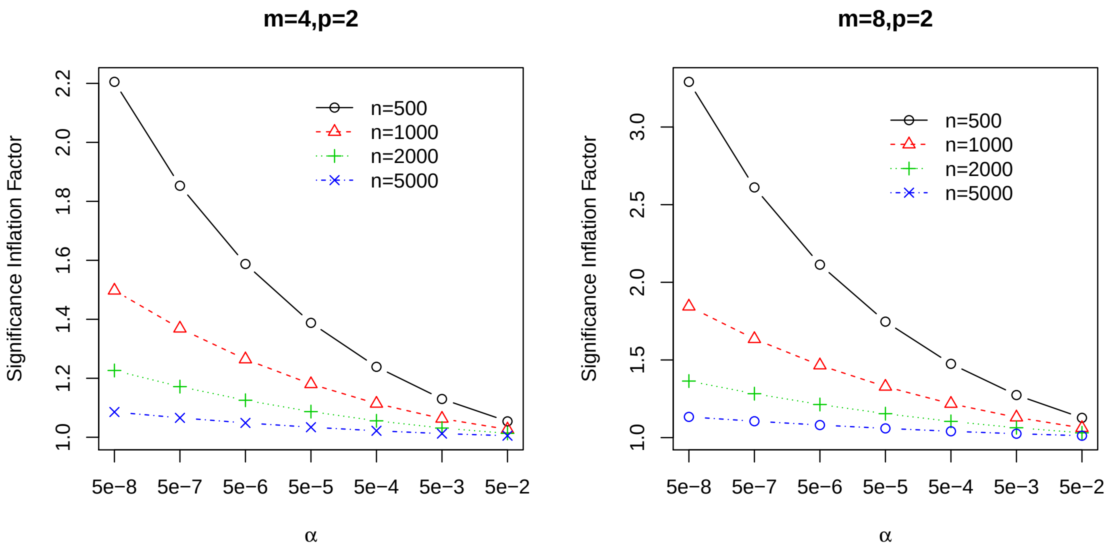

size almost increases linearly with number of traits m. For typical GWAS with small to medium sample sizes, we recommend using the appropriate F-distribution to compute significance p-values to reduce false positive findings.

4 Joint analysis of glycemic traits in ARIC GWAS

Table 1 lists the 62 genome-wide significant SNPs that were identified in the ARIC joint association test of fasting glucose (FG), fasting insulin (FI) and 2 hour fasting glucose levels (2hFG), and were also significant in the MAGIC consortium meta-analyses of these three traits (Dupuis *et al.*, 2010; Saxena *et al.*, 2010). In Table 1, we listed the ARIC joint test p-values (the proposed MLM Wald test and the GEE chi-square test), and the corresponding MAGIC consortium meta-analyses p-values.

Figure 2: Minimum sample size required to control IF ≤ 1.1 at significance level $\alpha = 5 \times 10^{-8}$: assume testing m quantitative traits with $p = 2$ covariates.

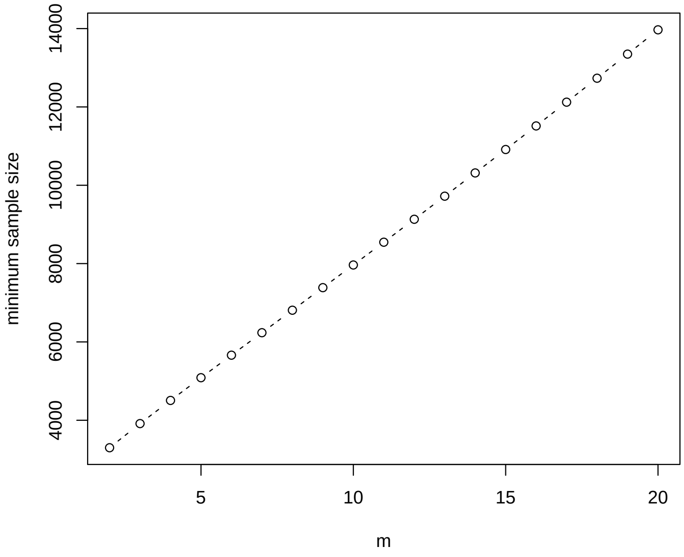

Table 2 lists the 79 novel SNPs that were identified in the ARIC joint association test of FG, IS and 2hFG, but have not been reported as significantly associated with diabetes related fasting glucose and insulin levels before. Among them, one SNP rs4665987 is located on chromosome 2:27755825 and another 78 SNPs are clustered on chromosome 15:62132921 to 15:62396389. Majority of them have large meta-analysis p-values for FG and FI, and relatively small p-values (around 10^{-5}) for the 2hFG. Interestingly 6 of them (rs4502156, rs7163757, rs8037894, rs6494307, rs7167878, rs7172432) were genome-wide significant in the MAGIC meta analysis of fasting proinsulin level (FP, Strawbridge *et al.*, 2011), with meta-analysis p-values ranging from 3.8×10^{-11} to 8.7×10^{-11}. We have highlighted these 6 SNPs in yellow at Table 2. We also provided the corresponding MAGIC meta-analysis p-values for FG, FI, 2hFG, and FP.

Table 1: Genome-wide significant SNPs that were identified in the ARIC joint association test of FG (fasting glucose), FI (fasting insulin) and 2hFG (2 hour fasting glucose), and were also significant in the MAGIC consortium meta-analyses.

			ARIC joint test Pval		MAGIC meta-analysis Pval		
SNP	Chr	bp	Wald	GEE	FG	FI	2hFG
rs1260326	2	27730940	1.0E-09	2.7E-09	4.3E-13	1.2E-04	1.5E-06
rs780094	2	27741237	1.4E-10	3.1E-10	2.5E-12	9.8E-05	1.5E-06
rs780093	2	27742603	1.5E-10	3.2E-10	2.9E-13	2.0E-04	1.7E-06
rs13431652	2	169753415	3.8E-11	3.5E-11	2.9E-63	1.4E-01	7.8E-01
rs1402837	2	169757354	1.8E-08	1.9E-08	7.4E-40	1.2E-01	7.7E-01
rs573225	2	169757541	1.2E-11	1.4E-11	6.2E-71	1.4E-01	6.0E-01
rs560887	2	169763148	3.0E-13	3.8E-13	4.6E-75	6.2E-02	4.1E-01
rs563694	2	169774071	1.6E-13	2.5E-13	1.2E-71	1.2E-01	1.5E-01
rs537183	2	169774646	1.5E-13	2.4E-13	9.0E-73	1.1E-01	1.6E-01
rs502570	2	169774959	1.5E-13	2.4E-13	9.2E-73	1.1E-01	1.6E-01
rs475612	2	169776746	6.7E-13	7.7E-13	1.0E-65	8.0E-02	1.9E-01
rs557462	2	169777595	1.5E-13	2.3E-13	3.4E-72	1.1E-01	1.6E-01
rs478333	2	169779156	2.6E-08	2.7E-08	3.2E-36	8.2E-02	3.9E-01
rs496550	2	169779712	2.6E-08	2.7E-08	1.3E-36	8.7E-02	4.3E-01
rs473351	2	169779896	1.1E-09	1.8E-09	5.7E-43	1.1E-01	1.8E-01
rs575671	2	169780818	1.1E-09	1.8E-09	5.5E-43	1.1E-01	2.0E-01
rs519887	2	169780885	2.5E-08	2.6E-08	2.6E-36	7.3E-02	5.2E-01
rs486981	2	169782149	1.1E-13	2.2E-13	2.5E-67	1.1E-01	2.9E-01
rs484066	2	169782481	7.7E-12	9.7E-12	1.4E-59	6.9E-02	5.1E-01
rs569805	2	169782880	1.1E-13	2.2E-13	2.6E-67	1.1E-01	3.0E-01
rs579060	2	169783039	1.1E-13	2.2E-13	2.5E-67	1.2E-01	3.0E-01
rs17540154	2	169784493	7.3E-08	4.2E-08	8.7E-38	1.0E-01	7.2E-01
rs508506	2	169784955	1.1E-13	2.2E-13	2.2E-67	1.2E-01	3.0E-01
rs503931	2	169785449	2.4E-08	2.6E-08	5.5E-36	7.7E-02	5.1E-01
rs551754	2	169787686	2.6E-08	2.7E-08	2.4E-36	8.4E-02	5.0E-01
rs497692	2	169789016	3.2E-08	3.3E-08	1.9E-35	7.4E-02	5.0E-01
rs494874	2	169789306	2.2E-13	3.6E-13	3.3E-67	1.3E-01	3.4E-01
rs552976	2	169791438	5.4E-13	7.6E-13	7.1E-66	9.6E-02	3.0E-01
rs567074	2	169794431	6.1E-09	6.4E-09	2.3E-42	1.0E-01	6.3E-01
rs2544367	2	169796288	3.5E-08	3.6E-08	2.1E-37	7.3E-02	6.4E-01
rs2685805	2	169797060	3.5E-08	3.6E-08	3.9E-37	7.7E-02	6.4E-01
rs1581397	2	169797652	3.4E-08	3.5E-08	1.5E-37	6.7E-02	6.4E-01
rs2685814	2	169798619	3.2E-08	3.4E-08	1.3E-37	7.3E-02	6.2E-01
rs853789	2	169801488	1.2E-14	2.5E-14	1.9E-67	9.8E-02	2.9E-01
rs860510	2	169801628	3.1E-08	3.2E-08	5.2E-38	6.0E-02	6.6E-01
rs853788	2	169801905	3.1E-08	3.2E-08	1.6E-38	6.3E-02	6.6E-01
rs853787	2	169802252	1.2E-14	2.5E-14	3.7E-73	9.9E-02	3.1E-01
rs853786	2	169802310	3.1E-08	3.2E-08	2.3E-38	6.0E-02	6.7E-01
rs862662	2	169802329	2.0E-09	2.3E-09	6.7E-44	6.3E-02	6.0E-01

rs853785	2	169802594	3.1E-08	3.2E-08	1.1E-38	6.0E-02	6.6E-01
rs853784	2	169803674	3.5E-08	4.0E-08	8.3E-39	4.8E-02	4.7E-01
rs853783	2	169805511	4.0E-08	4.5E-08	1.4E-38	4.5E-02	4.7E-01
rs853781	2	169806321	2.7E-09	3.3E-09	5.9E-44	4.6E-02	4.3E-01
rs853780	2	169807482	4.9E-08	5.5E-08	2.1E-38	5.4E-02	4.8E-01
rs1101533	2	169808522	3.9E-08	4.1E-08	1.0E-38	4.8E-02	4.6E-01
rs853779	2	169809672	3.3E-08	3.4E-08	1.5E-36	2.7E-02	4.6E-01
rs853778	2	169811224	5.3E-09	5.3E-09	1.1E-39	2.5E-02	4.2E-01
rs853773	2	169814347	1.1E-08	1.9E-08	4.8E-51	4.0E-02	7.9E-01
rs3847554	11	92668826	3.4E-09	2.6E-09	6.9E-39	6.6E-01	1.0E-01
rs12792753	11	92668975	4.8E-09	3.0E-09	2.7E-46	9.2E-01	1.4E-01
rs7112766	11	92672021	8.9E-15	1.6E-14	1.3E-43	7.4E-01	1.4E-02
rs1387153	11	92673828	2.2E-15	7.7E-15	6.6E-45	4.4E-01	1.4E-02
rs11523890	11	92679778	5.2E-10	4.2E-10	9.6E-51	8.6E-01	6.9E-02
rs10830956	11	92681013	1.9E-15	6.8E-15	8.6E-50	7.1E-01	1.3E-02
rs10765573	11	92683332	3.6E-10	2.8E-10	1.2E-51	8.3E-01	5.2E-02
rs7933855	11	92684322	1.1E-09	8.3E-10	1.5E-45	7.8E-01	3.0E-02
rs7936247	11	92690032	2.8E-10	2.2E-10	5.1E-52	9.1E-01	4.6E-02
rs11020124	11	92690661	1.2E-14	2.7E-14	5.1E-50	7.3E-01	1.8E-02
rs2166706	11	92691532	1.0E-09	7.8E-10	4.1E-44	8.3E-01	2.4E-02
rs10830961	11	92694757	1.9E-11	1.4E-11	1.7E-52	8.6E-01	2.3E-02
rs10830962	11	92698427	2.0E-11	1.5E-11	3.2E-51	9.5E-01	2.5E-02
rs10830963	11	92708710	6.3E-26	1.6E-24	1.3E-68	4.0E-01	1.2E-02

Table 2: 79 novel SNPs identified in the ARIC joint association test of FG, FI and 2hFG. The corresponding MAGIC consortium meta-analyses p-values for the three traits together with the FP (fasting proinsulin) are also listed.

			ARIC joint test Pval		MAGIC meta-analysis Pval			
SNP	Chr	bp	Wald	GEE	FG	FI	2hFG	FP
rs4665987	2	27755825	4.9E-08	1.0E-07	4.5E-06	3.7E-02	9.3E-05	6.9E-02
rs17271144	15	62132921	2.5E-08	1.7E-08	6.8E-02	8.4E-01	9.5E-06	5.2E-04
rs3743297	15	62149784	1.5E-08	9.1E-09	3.3E-02	8.4E-01	2.0E-05	7.0E-04
rs12908081	15	62162264	1.6E-08	1.0E-08	4.0E-02	8.7E-01	2.0E-05	9.1E-04
rs1981916	15	62171479	9.1E-09	5.4E-09	3.6E-02	9.9E-01	1.3E-05	8.1E-04
rs2414755	15	62172429	9.1E-09	5.4E-09	4.1E-02	9.9E-01	1.6E-05	7.0E-04
rs2414753	15	62200974	1.1E-08	5.6E-09	6.3E-02	8.3E-01	6.5E-06	4.6E-04
rs963024	15	62211450	2.9E-08	1.6E-08	6.5E-02	9.6E-01	3.3E-05	5.4E-04
rs4775453	15	62217391	1.5E-08	8.8E-09	7.2E-01	6.7E-01		4.2E-04
rs4774427	15	62217444	1.2E-08	6.0E-09	9.5E-02	9.8E-01	8.3E-06	5.3E-04
rs7172967	15	62218568	1.2E-08	6.0E-09	6.6E-02	8.7E-01	8.4E-06	5.3E-04
rs12439934	15	62224613	1.9E-08	1.4E-08	8.8E-02	9.6E-01	1.5E-05	2.7E-04
rs11071642	15	62229356	1.3E-08	6.6E-09	7.3E-02	9.1E-01	7.6E-06	5.8E-04
rs2042608	15	62232380	2.7E-09	2.0E-09	7.7E-02	9.8E-01	7.2E-06	1.9E-04

rs8033816	15	62233167	1.9E-08	1.5E-08	7.4E-02	7.9E-01	1.3E-05	4.1E-04
rs7170293	15	62236373	5.0E-09	3.1E-09	4.6E-02	9.9E-01	2.2E-05	4.3E-04
rs7177173	15	62236804	6.0E-09	3.2E-09	9.2E-02	9.8E-01	1.3E-05	3.1E-04
rs1425270	15	62237710	3.9E-09	2.5E-09	2.0E-02	9.1E-01	1.5E-05	4.2E-04
rs7166891	15	62239304	5.5E-09	3.3E-09	5.1E-02	9.9E-01	2.4E-05	4.4E-04
rs7172145	15	62239697	5.8E-09	3.5E-09	5.0E-02	1.0E+00	2.5E-05	4.8E-04
rs4587915	15	62241962	2.8E-09	1.7E-09	2.6E-02	9.2E-01	1.5E-05	5.4E-04
rs12899801	15	62246864	4.0E-08	3.2E-08	1.1E-01	7.9E-01	1.7E-05	2.8E-04
rs12593844	15	62247067	4.0E-08	3.1E-08	1.4E-01	8.6E-01	2.1E-05	2.8E-04
rs8027751	15	62247720	4.4E-09	2.2E-09	1.1E-01	9.7E-01	1.4E-05	3.7E-04
rs12910541	15	62248911	4.6E-08	3.0E-08	8.7E-02	9.3E-01	1.3E-05	3.0E-04
rs5006593	15	62250008	6.5E-09	3.4E-09	9.0E-02	9.9E-01	2.0E-05	3.3E-04
rs8034914	15	62250430	6.5E-09	3.4E-09	8.9E-02	9.7E-01	2.0E-05	3.4E-04
rs1344601	15	62259066	1.3E-08	8.2E-09	1.2E-01	9.2E-01	4.3E-06	3.3E-04
rs3784634	15	62259637	2.8E-09	1.4E-09	6.9E-02	9.6E-01	8.6E-06	4.5E-04
rs3784633	15	62259772	2.0E-08	1.4E-08	1.1E-01	9.5E-01	6.0E-06	2.8E-04
rs933807	15	62274940	1.4E-08	9.1E-09	1.3E-01	9.7E-01	3.0E-06	4.1E-04
rs2162062	15	62279937	1.3E-08	8.4E-09	1.2E-01	9.7E-01	3.4E-06	3.8E-04
rs11639482	15	62282114	1.1E-08	7.2E-09	1.2E-01	9.8E-01	3.6E-06	3.8E-04
rs8032433	15	62284101	1.6E-08	1.1E-08	1.2E-01	9.4E-01	3.5E-06	3.2E-04
rs8034335	15	62287456	1.1E-09	5.5E-10	8.9E-02	9.1E-01	4.9E-06	8.0E-04
rs8034216	15	62287528	1.1E-09	5.5E-10	6.5E-02	9.5E-01	4.1E-06	5.4E-04
rs12594658	15	62305090	5.6E-09	3.0E-09	1.2E-01	9.1E-01	2.2E-06	2.4E-04
rs17271305	15	62332980	1.0E-09	7.1E-10	2.2E-02	7.9E-01	1.0E-06	3.9E-03
rs1436958	15	62338797	1.3E-08	1.1E-08	4.9E-02	6.9E-01	6.2E-07	2.5E-03
rs17205365	15	62340126	3.0E-08	2.7E-08	2.5E-01	3.8E-01	5.2E-07	8.0E-04
rs12442675	15	62346056	8.0E-09	4.2E-09	1.7E-01	8.2E-01	6.1E-06	4.2E-04
rs4775455	15	62347274	8.1E-09	4.2E-09	1.3E-01	8.4E-01	9.0E-06	3.5E-04
rs17271340	15	62347885	8.8E-10	4.0E-10	7.4E-02	8.8E-01	2.3E-05	8.1E-04
rs12592402	15	62349020	8.1E-09	4.2E-09	1.3E-01	8.4E-01	9.6E-06	3.6E-04
rs8029942	15	62353458	1.2E-08	7.1E-09	6.0E-02	8.2E-01	2.7E-05	3.7E-04
rs12912208	15	62354570	1.2E-08	7.2E-09	9.8E-01	9.2E-01		4.2E-04
rs1436966	15	62358682	1.2E-08	6.8E-09	6.6E-02	8.0E-01	2.7E-05	3.8E-04
rs8039105	15	62359085	1.1E-09	4.5E-10	7.8E-02	8.3E-01	2.8E-05	1.0E-03
rs8039651	15	62359350	9.3E-09	4.5E-09	8.2E-01	8.9E-01		5.0E-04
rs1030859	15	62364932	6.2E-09	2.8E-09	1.1E-01	7.6E-01	1.3E-05	3.1E-04
rs11635977	15	62370484	3.5E-09	1.5E-09	1.1E-01	7.8E-01	7.7E-06	5.9E-04
rs11633500	15	62372475	3.5E-09	1.5E-09	1.1E-01	8.0E-01	6.3E-06	5.9E-04
rs12913951	15	62372592	3.5E-09	1.5E-09	1.1E-01	9.9E-01	4.3E-06	6.1E-04
rs17205407	15	62372827	3.5E-09	1.5E-09	8.8E-02	7.6E-01	5.4E-06	5.4E-04
rs7162536	15	62373459	3.5E-09	1.5E-09	1.3E-01	9.2E-01	1.1E-05	4.9E-04
rs1436964	15	62374487	3.6E-09	1.5E-09	1.1E-01	7.9E-01	6.6E-06	6.2E-04
rs4775458	15	62375036	3.9E-09	1.6E-09	1.2E-01	7.8E-01	6.8E-06	6.4E-04
rs17271403	15	62375389	3.7E-09	1.5E-09	8.5E-02	8.6E-01	8.5E-06	4.9E-04
rs8026008	15	62377805	4.6E-09	2.0E-09	1.2E-01	7.7E-01	8.4E-06	7.4E-04

rs8025877	15	62377820	4.8E-09	2.0E-09	1.2E-01	7.7E-01	9.3E-06	7.5E-04
rs893158	15	62378608	5.1E-09	2.1E-09	1.2E-01	7.6E-01	1.3E-05	8.0E-04
rs893156	15	62378892	5.7E-09	2.4E-09	1.3E-01	7.7E-01	1.2E-05	8.6E-04
rs7177276	15	62379668	5.9E-09	2.5E-09	1.3E-01	7.8E-01	1.2E-05	9.0E-04
rs7178945	15	62379814	6.2E-09	2.6E-09	1.3E-01	7.7E-01	1.2E-05	9.1E-04
rs7177711	15	62379971	6.6E-09	2.8E-09	1.3E-01	7.7E-01	1.2E-05	9.2E-04
rs7178540	15	62380132	7.0E-09	3.0E-09	1.3E-01	7.5E-01	1.2E-05	1.1E-03
rs7178424	15	62380259	6.9E-09	2.9E-09	1.4E-01	6.8E-01	1.3E-05	9.0E-04
rs12442212	15	62380482	6.6E-09	2.7E-09	1.1E-01	8.0E-01	1.9E-05	7.6E-04
rs12439356	15	62380595	6.9E-09	2.8E-09	1.3E-01	6.7E-01	1.7E-05	1.0E-03
rs17271431	15	62381016	6.8E-09	2.8E-09	1.3E-01	7.7E-01	1.5E-05	8.6E-04
rs17271438	15	62381065	6.3E-09	2.8E-09	1.2E-01	7.7E-01	1.5E-05	8.4E-04
rs17205463	15	62381413	6.1E-09	2.7E-09	1.2E-01	7.6E-01	1.5E-05	8.5E-04
rs10519157	15	62381630	9.2E-09	4.6E-09	1.3E-01	6.5E-01	4.8E-05	2.9E-03
rs4502156	15	62383155	5.4E-09	7.9E-09	8.4E-08	6.7E-01	8.2E-05	3.8E-11
rs7163757	15	62391608	1.4E-08	1.8E-08	4.2E-07	5.7E-01	1.9E-05	3.9E-11
rs8037894	15	62394264	1.2E-08	1.6E-08	4.1E-07	4.8E-01	3.5E-05	8.7E-11
rs6494307	15	62394690	1.7E-08	2.1E-08	3.3E-07	4.9E-01	2.7E-05	4.1E-11
rs7167878	15	62396189	1.7E-08	2.1E-08	4.6E-07	4.5E-01	2.4E-05	4.1E-11
rs7172432	15	62396389	1.7E-08	2.2E-08	6.5E-07	3.3E-01	1.9E-05	4.3E-11

5 Multivariate trait association test with different covariates

For trait y_k, denote x_k as its covariate vector (including the intercept) of length p_k, $k = 1, \ldots, m$. Here x_k are different across traits. Assume the multivariate regression model, $y_k = x_k^T \beta_{0k} + G\beta_{1k} + \epsilon_k$, $k = 1, \ldots, m$, where β_{0k} is of length p_k, and $\boldsymbol{\epsilon} = (\epsilon_1, \ldots, \epsilon_m)^T$ follows a zero-mean multivariate normal distribution with covariance Σ, $\boldsymbol{\epsilon} \sim N(0, \Sigma)$. This model is also known as seemingly unrelated regression (SUR) model or multiple-design multivariate (MDM) model (see, e.g., Timm, 2002, chapter 5).

Given observations of n individuals, denote the $n \times m$ response matrix as $\boldsymbol{Y}$, the $m \times p$ block matrix as $\boldsymbol{F}_i = \text{diag}(F_{i1}^T, \ldots, F_{im}^T) = \oplus_{k=1}^m F_{ik}^T$ for the i-th individual, where $p = \sum_{k=1}^m (p_k + 1)$ and $F_{ik} = (x_{ik}^T, G_i)^T$ is a column vector of length $p_k + 1$, and the $n \times m$

error matrix as $\boldsymbol{B} = (\boldsymbol{\epsilon}_1, \ldots, \boldsymbol{\epsilon}_n)^T$. Denote the $(nm) \times p$ matrix $\boldsymbol{A} = (\boldsymbol{F}_1^T, \ldots, \boldsymbol{F}_n^T)^T$, and $\boldsymbol{\beta} = (\beta_{01}^T, \beta_{11}, \ldots, \beta_{0m}^T, \beta_{1m})^T$ of length p. Then the SUR or MDM model can be written in matrix notation as $vec(\boldsymbol{Y}^T) = \boldsymbol{A}\boldsymbol{\beta} + vec(\boldsymbol{B}^T)$. The MLEs can be very efficiently solved based on iteration of $\hat{\boldsymbol{\beta}} = [\boldsymbol{A}^T(\boldsymbol{I}_n \otimes \Sigma^{-1})\boldsymbol{A}]^{-1}[\boldsymbol{A}^T(\boldsymbol{I}_n \otimes \Sigma^{-1})vec(\boldsymbol{Y}^T)]$ and updating $\hat{\Sigma}$ as the sample covariance of residuals (see, e.g., Timm, 2002, chapter 5). Note that $Cov(\hat{\boldsymbol{\beta}}) = [\boldsymbol{A}^T(\boldsymbol{I}_n \otimes \Sigma^{-1})\boldsymbol{A}]^{-1}$. When computing the Wald statistics, we plug in the estimated $\hat{\Sigma}$ and account for its estimation uncertainty by using an approximate F-distribution to compute p-values (see, e.g., Timm, 2002, p. 313). Here $\boldsymbol{A}^T(\boldsymbol{I}_n \otimes \Sigma^{-1})\boldsymbol{A} = \sum_{i=1}^n \boldsymbol{F}_i^T \Sigma^{-1} \boldsymbol{F}_i$, $\boldsymbol{A}^T(\boldsymbol{I}_n \otimes \Sigma^{-1})vec(\boldsymbol{Y}^T) = \sum_{i=1}^n \boldsymbol{F}_i^T \Sigma^{-1} \mathbf{Y}_i$.

5.1 Simulation study

We consider a common Bernoulli covariate Z with probability of 0.5 (population indicator), and separately simulate a standard normal covariate X_k for each trait Y_k. The SNP genotype score G is simulated from a Binomial distribution, Binom(2,f_0), where the minor allele frequency (MAF) $f_0 = p_0 + p_1 Z$.

We conducted simulations for testing $m = 2, 4, 8$ related traits of 1,000 unrelated individuals respectively. Each time we simulate the m traits from a multivariate normal distribution with a compound symmetry correlation matrix with correlation ρ. The first trait has a variance of 2 and all the other traits have unit variance. We set $E(Y_i) = 1 + 0.5X_i + 0.5Z + \gamma_i G$ for $i = 1, \ldots, m-1$, and $E(Y_k) = 1 + X_k + Z + \gamma_k G$ for $k = 2, \ldots, m$.

We used 10 million experiments to evaluate the type I error, and 10^5 experiments to evaluate the power under various combinations of $(\gamma_1, \ldots, \gamma_m)$. We conducted simulations for $p_0 = (0.1, 0.3)$, $p_1 = 0.1$, and $\rho = 0, 0.2, 0.5, 0.8$. Here we report the results for $m = 2, 8$, $\rho = 0, 0.5$ and $p_0 = 0.1$. The conclusions remain the same for other settings (data not shown).

Table 3: Type I error of testing two continuous traits, scaled by the nominal significance level α. The MAFs of SNP are 0.1 and 0.2 in the two populations. Q is the m-DF omnibus Wald test, T and T' are the 1-DF Wald tests assuming a common or common scaled effect. (Q_s, T_s, T_s') are the corresponding MLM GEE based m-DF omnibus test and 1-DF tests assuming a common effect or common scaled effect. $(\tilde{Q}, \tilde{T}, \tilde{T}')$ are the Wald tests using chi-square distribution to compute p-values.

$\rho = 0$									
α	Q	T	T'	$\tilde{Q}$	$\tilde{T}$	$\tilde{T}'$	Q_s	T_s	T_s'
10^{-5}	1.03	1.06	1.08	1.19	1.16	1.18	0.69	0.86	0.87
10^{-4}	1.02	1.02	1.03	1.11	1.11	1.12	0.81	0.85	0.89
10^{-3}	1.00	1.00	1.01	1.05	1.04	1.06	0.89	0.94	0.94
$\rho = 0.5$									
α	Q	T	T'	$\tilde{Q}$	$\tilde{T}$	$\tilde{T}'$	Q_s	T_s	T_s'
10^{-5}	1.07	0.96	1.06	1.15	1.08	1.10	0.66	0.66	0.83
10^{-4}	1.04	1.00	1.00	1.14	1.08	1.07	0.82	0.87	0.91
10^{-3}	0.99	0.99	1.00	1.05	1.03	1.04	0.90	0.93	0.93

Tables 3 and 4 summarize the estimated type I errors. Overall the type I errors are well controlled for the proposed methods, while the GEE score tests are conservative especially for large number of traits ($m = 8$).

Tables 5 and 6 summarize the power for $m = 2$ and $m = 8$ respectively. T is the most powerful when γ_j are close to each other, and T' is the most powerful when γ_j/σ_j are close to each other. In general the proposed MLM based Wald tests perform better than the corresponding GEE based score tests. This agrees with the general principle that the Wald test is typically more powerful than the GEE based test.

Table 4: Type I error of testing eight continuous traits, scaled by the nominal significance level α. The MAFs of SNP are 0.1 and 0.2 in the two populations.

α	Q	T	T'	$\tilde{Q}$	$\tilde{T}$	$\tilde{T}'$	Q_s	T_s	T'_s
$\rho = 0, p_0 = 0.1$									
10^{-5}	0.84	1.07	1.05	1.11	1.25	1.15	0.48	0.85	0.78
10^{-4}	0.88	0.99	1.00	1.11	1.10	1.11	0.60	0.86	0.85
10^{-3}	0.93	1.01	1.00	1.11	1.08	1.09	0.75	0.95	0.94
$\rho = 0.5, p_0 = 0.1$									
10^{-5}	0.92	0.78	0.93	1.33	0.98	1.05	0.42	0.62	0.65
10^{-4}	0.94	0.96	0.99	1.24	1.09	1.12	0.64	0.90	0.86
10^{-3}	0.95	0.95	1.00	1.13	1.02	1.07	0.76	0.94	0.94
$\rho = 0, p_0 = 0.3$									
10^{-5}	1.00	0.96	1.03	1.42	1.15	1.17	0.66	0.86	0.92
10^{-4}	0.90	0.96	0.97	1.17	1.07	1.10	0.77	0.90	0.90
10^{-3}	0.93	1.01	1.02	1.11	1.09	1.10	0.84	0.97	0.97
$\rho = 0.5, p_0 = 0.3$									
10^{-5}	0.80	0.82	0.87	1.28	1.01	1.05	0.47	0.75	0.72
10^{-4}	0.91	0.88	1.00	1.16	0.99	1.15	0.73	0.88	0.95
10^{-3}	0.92	0.94	1.00	1.09	1.01	1.06	0.84	0.97	0.97

Table 5: Power of multi-trait tests for $m = 2$ continuous traits (Y_1, Y_2) under significance level $\alpha = 10^{-4}$. The MAFs of SNP are 0.1 and 0.2 in the two populations respectively. Q is the m-DF omnibus Wald test, T and T' are the 1-DF Wald tests assuming common or common scaled effect. (Q_s, T_s, T'_s) are the corresponding GEE based m-DF omnibus test and 1-DF tests assuming a common effect or common scaled effect. σ_i is the standard error of Y_i and γ_i is the SNP coefficient, $i = 1, 2$. The highest powered tests are bold-faced.

				$\rho = 0$			
(γ_1, γ_2)	$(\frac{\gamma_1}{\sigma_1}, \frac{\gamma_2}{\sigma_2})$	Q	T	T$'$	Q$_s$	T$_s$	T$'_s$
(0.3,0)	(0.21,0)	**0.205**	0.025	0.064	0.178	0.020	0.052
(0.3,0.1)	(0.21,0.1)	0.316	0.249	**0.337**	0.278	0.217	0.302
(0.25,0.18)	(0.18,0.18)	0.418	0.509	**0.530**	0.374	0.470	0.494
(0.3,0.25)	(0.21,0.25)	0.831	0.891	**0.892**	0.796	0.869	0.870
(0.2,0.2)	(0.14,0.2)	0.376	**0.484**	0.462	0.335	0.449	0.426
(0.2,0.25)	(0.14,0.25)	0.631	**0.727**	0.676	0.585	0.694	0.638
(0.25,0.25)	(0.18,0.25)	0.731	**0.818**	0.799	0.690	0.791	0.769
(0,0.25)	(0,0.25)	**0.401**	0.247	0.133	0.359	0.216	0.108
(0,0.3)	(0,0.3)	**0.701**	0.486	0.291	0.657	0.439	0.239
(0.1,0.25)	(0.07,0.25)	0.463	**0.484**	0.372	0.418	0.448	0.331
(0.1,0.3)	(0.07,0.3)	**0.744**	0.726	0.590	0.701	0.690	0.535
(0.2,0.3)	(0.14,0.3)	0.842	**0.890**	0.842	0.810	0.869	0.809
				$\rho = 0.5$			
(0.3,0)	(0.21,0)	**0.377**	0.001	0.025	0.334	0.001	0.019
(0.3,0.1)	(0.21,0.1)	**0.208**	0.049	0.145	0.179	0.041	0.127
(0.25,0.18)	(0.18,0.18)	0.178	0.218	**0.255**	0.153	0.192	0.232
(0.3,0.25)	(0.21,0.25)	0.522	0.615	**0.617**	0.477	0.573	0.582
(0.2,0.2)	(0.14,0.2)	0.175	**0.255**	0.214	0.151	0.229	0.192
(0.2,0.25)	(0.14,0.25)	0.408	**0.498**	0.364	0.366	0.464	0.331
(0.25,0.25)	(0.18,0.25)	0.448	**0.558**	0.493	0.403	0.521	0.457
(0,0.25)	(0,0.25)	**0.639**	0.277	0.052	0.590	0.247	0.040
(0,0.3)	(0,0.3)	**0.890**	0.525	0.120	0.863	0.476	0.094
(0.1,0.25)	(0.07,0.25)	**0.451**	0.383	0.165	0.405	0.354	0.141
(0.1,0.3)	(0.07,0.3)	**0.771**	0.640	0.300	0.730	0.607	0.257
(0.2,0.3)	(0.14,0.3)	0.703	**0.746**	0.548	0.659	0.718	0.504

Table 6: Power of multi-trait tests for $m = 8$ continuous traits under significance level $\alpha = 10^{-4}$. The MAFs of SNP are 0.1 and 0.2 in the two populations respectively. Q is the m-DF omnibus Wald test, T and T' are the 1-DF Wald tests assuming common or common scaled effect. (Q_s, T_s, T_s') are the corresponding GEE based m-DF omnibus test and 1-DF tests assuming a common effect or common scaled effect. The highest powered tests are bold-faced.

$\rho = 0$						
$\gamma_1 = 0.3, \gamma_{i>1} = 0$	**0.063**	0.001	0.003	0.044	0.001	0.002
$(.3, .2, .1, .05, 0, \cdots, 0)$	**0.457**	0.153	0.219	0.373	0.101	0.152
$\gamma_1 = 0.2, \gamma_{i>1} = 0.15$	0.933	0.995	**0.996**	0.889	0.992	0.993
$\gamma_i = 0.15$	0.908	**0.994**	0.993	0.855	0.990	0.989
$\rho = 0.5$						
$(\gamma_1, \cdots, \gamma_8)$	Q	T	T$'$	Q$_s$	T$_s$	T$'_s$
$\gamma_1 = 0.3, \gamma_{i>1} = 0$	**0.297**	0.001	0	0.230	0	0
$(.3, .2, .1, .05, 0, \cdots, 0)$	**0.688**	0.001	0.008	0.596	0	0.005
$\gamma_1 = 0.2, \gamma_{i>1} = 0.15$	0.043	0.196	**0.217**	0.030	0.169	0.198
$\gamma_i = 0.15$	0.045	**0.230**	0.190	0.031	0.203	0.172

6 R package MTAR

We have implemented the proposed methods in an R package "MTAR" available at `http://www.github.com/baolinwu/MTAR`. The following lists some sample R codes to install and use the package.

```
## Install MTAR
  devtools::install_github("baolinwu/MTAR")
## Multi-trait association test
  library(VGAM)
  library(MTAR)
  Z = rbinom(1000,1,0.5)
  G = rbinom(1000,2,0.25)
  ## assume the same covariates
  X = rnorm(1000)
```

```
e = rnorm(1000)
Y1 = Z+X + 0.15*G + rnorm(1000)+e
Y2 = Z+X + 0.1*G + rnorm(1000)+e
Y = cbind(Y1,Y2)
obj = MLM.null(Y,cbind(Z,X))
MQTAc(obj, G)
## different covariates
X1 = rnorm(1000)
X2 = rnorm(1000)
Y1 = Z+X1 + 0.15*G + rnorm(1000)+e
Y2 = Z+X2 + 0.1*G + rnorm(1000)+e
Y = cbind(Y1,Y2)
YX = list(Y, cbind(X1,Z), cbind(X2,Z))
objd = MDM.null(YX,pux=2)
MQTAd(objd, G)
```

The developed algorithms are very efficient and extremely scalable to genome-wide association test. For example, it takes 30 minutes to conduct joint association tests for around 2.5 million HapMap SNPs in the ARIC data on a single Linux desktop with 3.0 GHz CPU and 24 GB memory. The eigen decompositions involved are computed very efficiently, since we just need to compute the top eigen vectors for the covariate matrix. The covariance matrix involved in the Wald tests has the same dimension as the number of traits, and its inverse can also be computed efficiently.

Table 7: Type I error (divided by the significance level α) when simulating from K-DF multivariate t-distribution.

	K=5			K=10			K=20		
α	Q	T	T'	Q	T	T'	Q	T	T'
10^{-4}	0.92	0.97	0.95	0.92	0.84	0.82	0.92	0.87	0.87
10^{-3}	0.95	0.98	0.98	1.01	0.99	0.99	0.97	0.96	0.98
10^{-2}	0.99	1.00	1.00	0.99	1.00	1.00	1.00	1.00	1.00

7 Discussions

We note that the multi-trait test approach generally benefits the most when the marginal effects have different directions from the trait correlations. For example, consider a bivariate normal random vector $Z = (z_1, z_2)^T$ with covariance matrix Σ having unit variance and 0.5 correlation. Consider the Wald test $Z^T\Sigma^{-1}Z$. Under 5×10^{-8} significance level, its test power is 0.5% with $E(z_1) = 3, E(z_2) = 2$, while the power is 24.8% when $E(z_1) = 3, E(z_2) = -2$.

The proposed Wald tests are generally robust to deviation from normality, partly because GWAS are based on large sample sizes, and the F-distribution based Wald test is robust. Here we conduct a simple simulation study to investigate the type I errors of the proposed Wald tests when we simulate the outcomes from the multivariate t-distribution instead. We consider 5000 individuals with two outcomes following K-DF bivariate t-distribution with unit variance and 0.5 correlation. The genotype is simulated from $\text{Binom}(2, 0.2)$. We conducted 10^6 simulations to investigate the type I errors at significance levels $\alpha = 10^{-2,-3,-4}$ under $K = 5, 10, 20$. Table 7 summarizes the results. Overall we can see that all proposed Wald tests have well controlled type I errors.

GWAS are typically useful to identify common variants (MAF$\geq$5%). The proposed Wald tests are applicable to any MAF and number of traits, in the sense that the Wald test F-distributions always hold. However we do recommend that the tests are only applied to

common variants and relatively small number of traits: for PheWAS with huge number of traits (Pendergrass *et al.*, 2011), more efforts are needed to develop new and powerful tests.

References

Dupuis,J., Langenberg,C., Prokopenko,I., Saxena,R., Soranzo,N., Jackson,A.U. and others. (2010) New genetic loci implicated in fasting glucose homeostasis and their impact on type 2 diabetes risk. *Nature Genetics,* **42** (2), 105–116.

Ferreira,M.A.R. and Purcell,S.M. (2009) A multivariate test of association. *Bioinformatics,* **25** (1), 132–133.

Pendergrass,S., Brown-Gentry,K., Dudek,S., Torstenson,E., Ambite,J., Avery,C., and others. (2011) The use of phenome-wide association studies (PheWAS) for exploration of novel genotype-phenotype relationships and pleiotropy discovery. *Genetic Epidemiology,* **35** (5), 410–422.

Purcell,S., Neale,B., Todd-Brown,K., Thomas,L., Ferreira,M.A.R., Bender,D., Maller,J., Sklar,P., de Bakker,P.I.W., Daly,M.J. and Sham,P.C. (2007) PLINK: a tool set for whole-genome association and population-based linkage analyses. *American Journal of Human Genetics,* **81** (3), 559–575.

Saxena,R., Hivert,M.F., Langenberg,C., Tanaka,T., Pankow,J.S., Vollenweider,P. and others. (2010) Genetic variation in GIPR influences the glucose and insulin responses to an oral glucose challenge. *Nature Genetics,* **42** (2), 142–148.

Strawbridge,R.J., Dupuis,J., Prokopenko,I., Barker,A., Ahlqvist,E., Rybin,D., and others. (2011) Genome-wide association identifies nine common variants associated with fasting

proinsulin levels and provides new insights into the pathophysiology of type 2 diabetes. *Diabetes,* **60** (10), 2624–2634.

Timm,N.H. (2002) *Applied Multivariate Analysis.* 2002 edition, Springer, New York.
